# CircRNA DDX21 acts as a prognostic factor and sponge of miR‐1264/QKI axis to weaken the progression of triple‐negative breast cancer

**DOI:** 10.1002/ctm2.768

**Published:** 2022-05-06

**Authors:** Renhong Huang, Zhou Yang, Qian Liu, Biao Liu, Xinyuan Ding, Zheng Wang

**Affiliations:** ^1^ Department of General Surgery Comprehensive Breast Health Center Ruijin Hospital Shanghai Jiao Tong University School of Medicine Shanghai China; ^2^ Department of General Surgery Shanghai Pudong Hospital Fudan University Pudong Medical Center Shanghai China; ^3^ Department of Pathology the Affiliated Suzhou Hospital of Nanjing Medical University Suzhou China; ^4^ Department of Pharmacy the Affiliated Suzhou Hospital of Nanjing Medical University Suzhou China

Dear Editor,

We report that circDDX21 functions as a sponge for miR‐1264 and regulates QKI expression, thus repressing the progression of triple‐negative breast cancer (TNBC). TNBC represents around approximately 15%–20% of breast cancer pathological types.^1^ Due to the lack of efficient targeted therapies, TNBC exhibits the characteristics of a relatively poor prognosis and a high recurrence rate and tends to show distant metastasis, which has become the focus of breast cancer research and attention in recent years.^2^

Circular RNAs (circRNAs) are a novel type of ncRNA that are mainly generated by the cyclisation of protein‐coding genes and other coding transcripts. In TNBC, circRNAs exhibit potentially powerful modulation of biological functions, such as tumour initiation, proliferation, invasion and metastasis. According to a previous study, circABCB10 could promote breast cancer progression by the sponging of miR‐1271.^3^ However, the potential roles of more circRNAs in the development of TNBC remain unclear.

In this study, we concentrated on a novel circRNA (Figures 1A and B, D–G, S1), circDDX21, which showed downregulated expression in TNBC tissues compared with normal breast tissues according to published datasets (Figure 1H).^4^ CircDDX21 expression was downregulated in the normal mammary gland cell line MCF10A compared with other BC cell lines (Figure 1I). We also confirmed that circDDX21 expression was remarkably downregulated in self‐collected TNBC tissues compared with non‐tumour breast tissues (Figure 1C, J). The expression of circDDX21 showed a close association with the tumour grade, lymph node metastasis, and pathological stage of TNBC (Table S1). Advanced TNBC showed significant downregulation of circDDX21 expression compared with early TNBC (Figure 1K, L). Interestingly, low expression of circDDX21 indicated prolonged survival in TNBC patients (Figure 1M, N).

**FIGURE 1:**
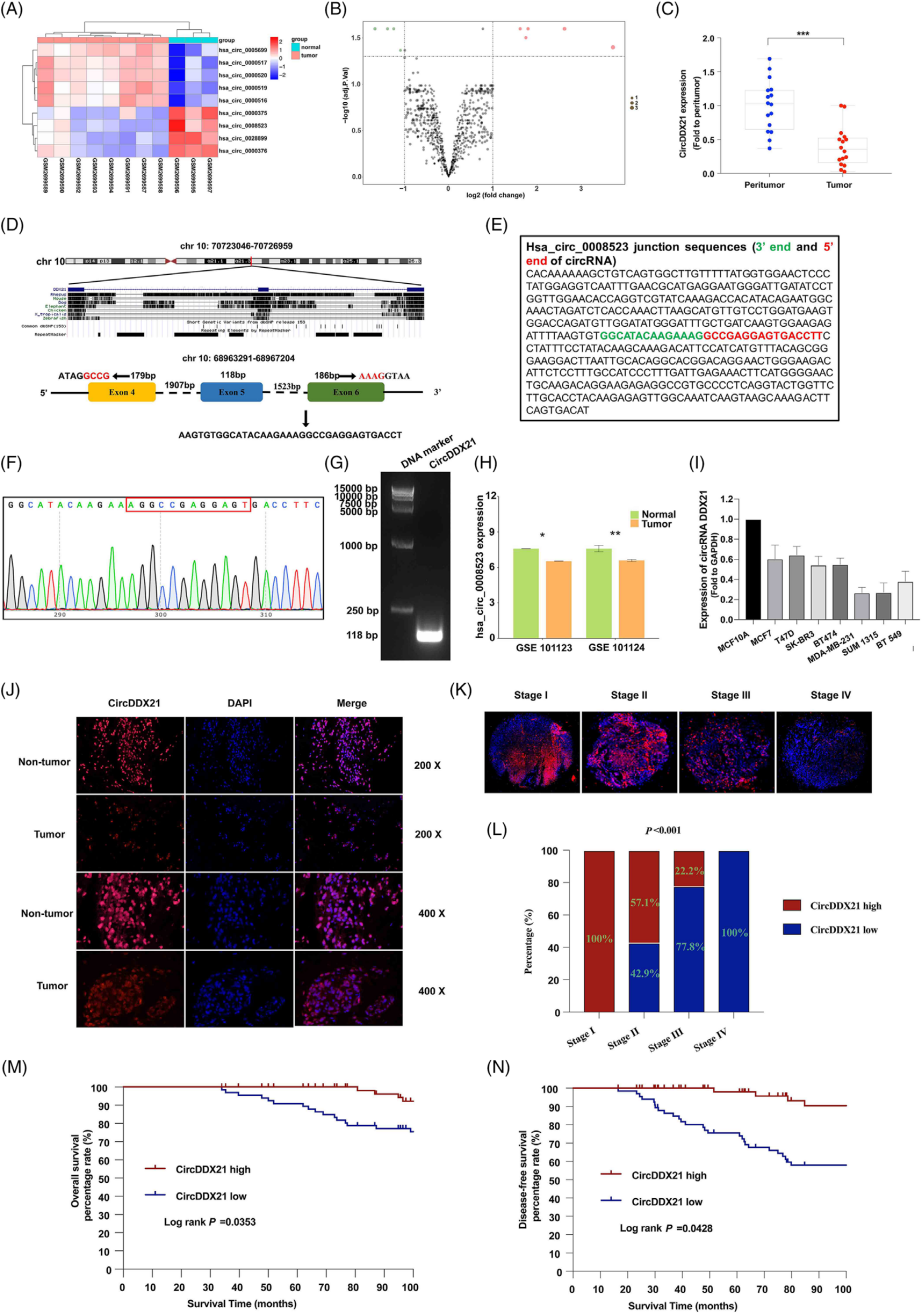
CircDDX21 is a prognostic factor for TNBC. (A) Heatmap of circDDX21 expression in the GSE101123 dataset. (B) Volcano plot of circDDX21 expression in the GSE101123 dataset (fold–change >  1 or  <  –1, *p*  <  .05). (C) The expression of circDDX21 in a cohort of self‐collected specimens was determined by RT‐qPCR. (D) Schematic illustration presenting the formation of circDDX21 from exons 4–6 of the DDX21 gene. (E–G) The specific primers of circDDX21 were validated by Sanger sequencing and agarose gel electrophoresis. (H) The expression of circDDX21 in the GSE101123 and GSE101124 datasets. (I) The expression of circDDX21 in the normal mammary gland cell line MCF10A and 7 other types of BC cell lines. (J) The expression of circDDX21 in self‐collected specimens determined by the FISH assay. (K and L) The expression of circDDX21 was associated with the pathological stage of TNBC. (M and N) The association of circDDX21 with overall survival and disease‐free survival in TNBC patients analysed by the Kaplan‐Meier method (^*^ *p*  < .05, ^**^ *p*  < .01, ^***^ *p*  < .001)

We overexpressed and repressed circDDX21 expression in 2 TNBC cell lines, SUM 1315 and MDA‐MB 231, to determine its role in TNBC (Figure 2A, B). Overexpression of circDDX21 repressed the proliferation, migration, invasion and clone formation abilities of the two TNBC cell lines, whereas circDDX21 knockdown promoted these abilities (Figures 2C–J, S3A–C). Similar results were also observed in the mouse TNBC cell line 4T1 (Figure S2).

**FIGURE 2:**
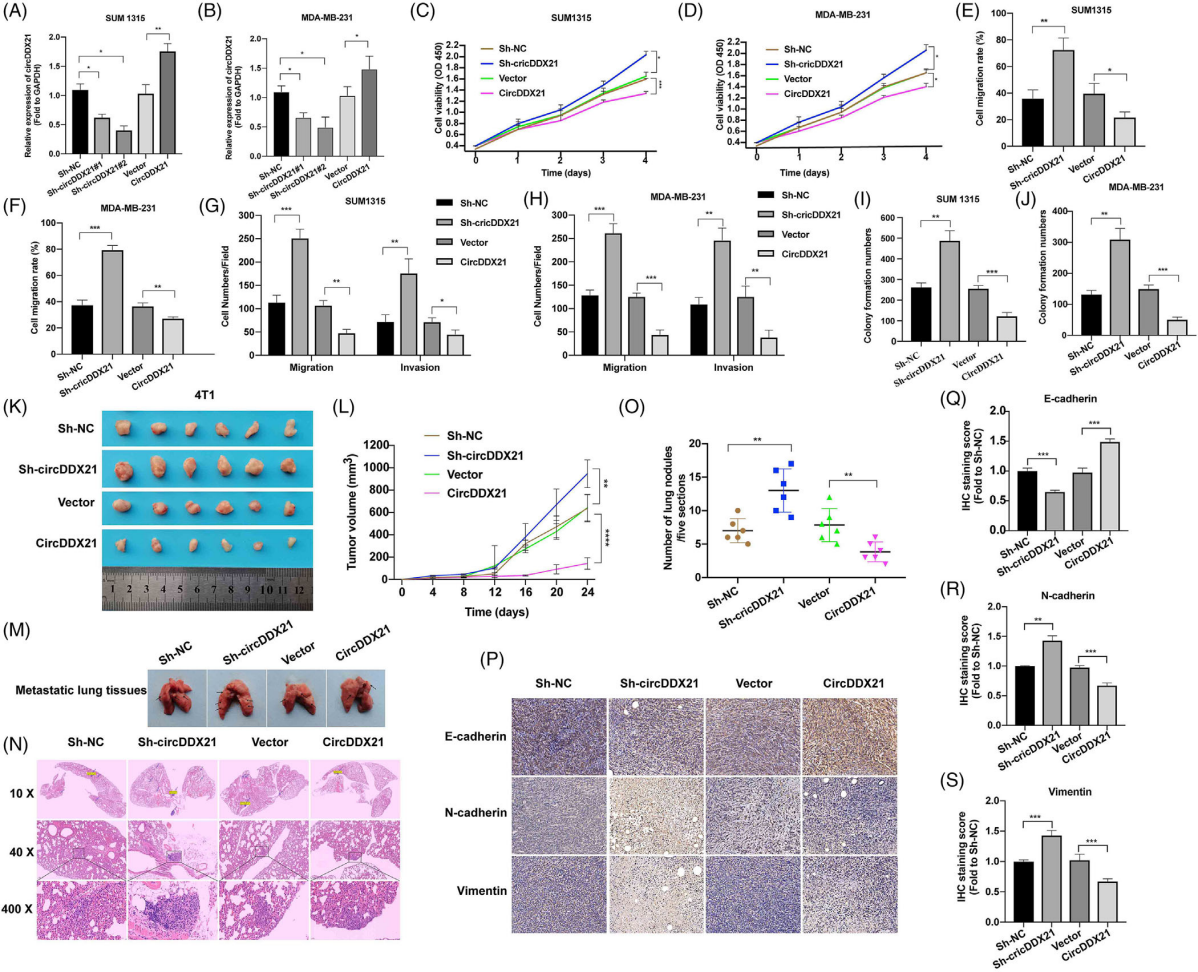
CircDDX21 regulates cell proliferation and invasion in vitro. (A and B) Overexpression and knockdown of circDDX21 in SUM 1315 and MDA‐MB‐231 cell lines were validated by RT‐qPCR. (C and D) The proliferation of SUM 1315 and MDA‐MB 231 cells after overexpression and knockdown of circDDX21. (E and F) The migration of SUM 1315 and MDA‐MB 231 cells was determined by a wound healing assay. (G and H) The migration and invasion of SUM 1315 and MDA‐MB 231 cells were determined by Transwell assays. (I and J) The clone formation ability of SUM 1315 and MDA‐MB 231 cells. (K and L) The homografts and growth curve derived from the circDDX21‐overexpressing and circDDX21‐downregulated 4T1 cells (*n* = 6 per group). (M–O) CircDDX21 regulated lung metastasis of 4T1 cells. (P–S) The expression of EMT‐associated proteins in xenografts (^*^ *p*  < .05, ^**^ *p*  < .01, ^***^ *p*  < .001)

Subsequently, we further investigated the role of circDDX21 in vivo. In a homograft mouse model, circDDX21 overexpression repressed growth, whereas circDDX21 knockdown promoted the growth of 4T1 cells in vivo (Figure 2K–L). In a lung metastasis model, circDDX21 overexpression inhibited metastasis, whereas circDDX21 knockdown promoted 4T1 cell metastasis in vivo (Figure 2M–O). We also measured the expression of EMT‐associated proteins in homografts using IHC. We found that circDDX21 overexpression promoted the expression of E‐cadherin, but inhibited N‐cadherin and Vimentin expression. In contrast, circDDX21 knockdown exerted the opposite effect (Figure 2P–S). In general, we confirmed that circDDX21 overexpression suppressed EMT, whereas circDDX21 knockdown promoted EMT. Similar results were also acquired with E0771 cell lines in vivo (Figure S4).

CircRNAs function as miRNA sponges, thus interacting with proteins and regulating transcription or translation. We successfully identified miR‐1264 as a direct target for circDDX21 by pull‐down and luciferase reporter assays (Figure 3A–E). We also confirmed that miR‐1264 showed significant co‐localisation with circDDX21 in the cell cytoplasm of TNBC cells (Figure 3F). We performed rescue assays to further understand the role of the circDDX21/miR‐1264 axis. Inhibition of miR‐1264 completely rescued the increased proliferation, migration, invasion and migration of circDDX21 knockdown cells (Figures 3G–P, S5A–C).

**FIGURE 3:**
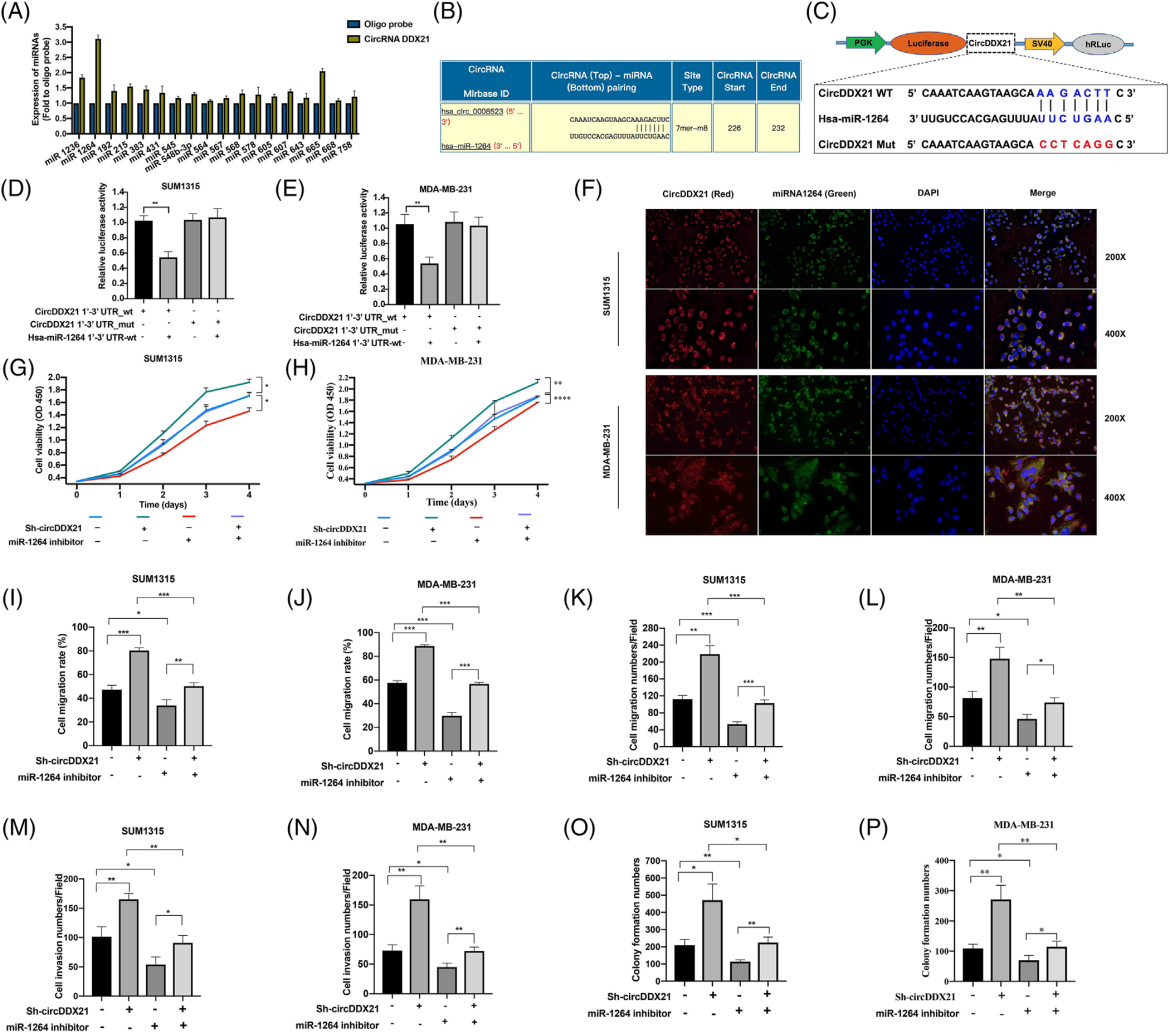
CircDDX21 acts as a sponge for miR‐1264. (A) RNA pull‐down assay using a biotin‐labelled circDDX21 probe. The expression of miRNAs was detected by RT‐qPCR. (B) Predicted binding site between circDDX21 and miR‐1264 in the miRBase database. (C) Construction of the circDDX21 luciferase reporter. (D and E) Luciferase reporter assays of TNBC cells transfected with miR‐1264 mimics and the wild type or mutant circDDX21 reporter. (F) Co‐localisation between circDDX21 and miR‐1264 performed by FISH assay. (G and H) The proliferation ability of the circDDX21 knockdown cells transfected with the miR‐1264 inhibitor. (I and J) The migration of circDDX21 knockdown cells transfected with the miR‐1264 inhibitor. (K–N) The migration and invasion of the circDDX21 knockdown cells transfected with the miR‐1264 inhibitor. (O–P) The clone formation ability of the circDDX21 knockdown cells transfected with the miR‐1264 inhibitor (^*^ *p*  < .05, ^**^ *p*  < .01, ^***^ *p*  < .001)

In previous study, miR‐1264 was confirmed to be a target of another non‐coding RNA, SOCS2‐AS1. SOCS2‐AS1 contributes to the expression of SOCS2 by restraining miR‐1264, thus inhibiting the progression of colorectal cancer.^5^ Subsequently, we further identified QKI as the direct downstream target of miR‐1264 by a luciferase reporter assay (Figure 4A–E). QKI is an RNA binding protein that has also been confirmed to be closely related to the development of various cancers. QKI expression is downregulated and associated with shorter survival of non‐small‐cell lung cancer (NSCLC) patients.^6^ QKI also regulates the progression of prostate cancer through the circZEB1/miR‐141‐3p/ZEB1 signalling pathway.^7^ Moreover, miR‐1264 overexpression repressed the expression of QKI, whereas downregulation of miR‐1264 expression promoted the expression of QKI (Figure 4F–H). In rescue assays, we found that QKI overexpression completely rescued the increased proliferation, migration, invasion and migration of miR‐1264 silenced cells (Figures 4I–R, S6A–C).

**FIGURE 4:**
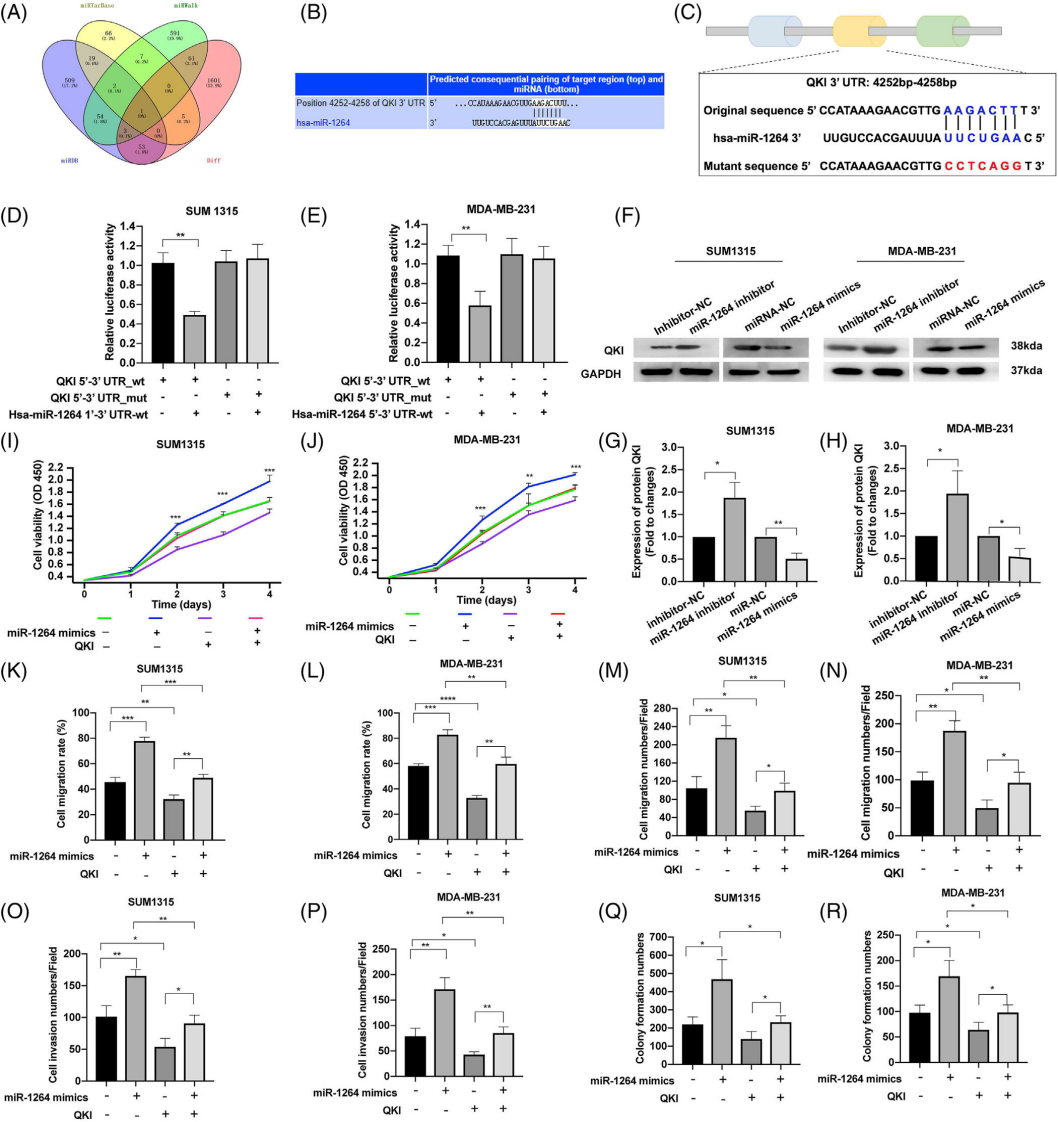
QKI is a direct target of miR‐1264. (A) Candidate genes predicted to be the potential targets of miR‐1264. (B) Predicted binding site between QKI and miR‐1264 in the miRBase database. (C) Construction of the QKI luciferase reporter. (D and E) Luciferase reporter assays of TNBC cells transfected with miR‐1264 mimics and the wild type or mutant QKI reporter. (F–H) The expression of QKI in the miR‐1264 inhibited and miR‐1264‐overexpressing TNBC cells. (I and J) The proliferation of the miR‐1264 inhibited cells transfected with QKI. (K and L) The migration of the miR‐1264 inhibited cells transfected with QKI. (M–P) The migration and invasion of the circDDX21 knockdown cells transfected with miR‐1264 inhibitor. (Q and R) The clone formation ability of the miR‐1264 inhibited cells transfected with QKI (^*^ *p*  < .05, ^**^ *p*  < .01, ^***^ *p*  < .001)

Thus far, we have confirmed that circDDX21 functions as a sponge for miR‐1264, to regulate the expression of QKI. Epithelial cell–mesenchymal transition (EMT) is the main process underlying the progression of cancers.^8^ Interestingly, DDX21 suppressed breast cancer metastasis by regulating EMT.^9^ Therefore, we wondered whether circDDX21 also regulates the EMT in TNBC. As expected, circDDX21 reduced the Vimentin and N‐cadherin expression but increased E‐cadherin expression, which is a classic characteristic of the EMT (Figure S7A–D). Similar results were also obtained using IF staining (Figure S7E–H). Interestingly, miR‐1264 inhibition rescued the changes in the levels of these EMT‐associated proteins in circDDX21 knockdown cells (Figure S8). Overexpression of miR‐1264 promoted the EMT process. Moreover, overexpression of QKI rescued the changes in the levels of these EMT‐associated proteins in miR‐1264‐overexpressing cells (Figure S9). Furthermore, we demonstrated that QKI regulates cell proliferation via the inhibition of the p38 MAPK signalling pathway (Figure S10). In general, circDDX21 repressed the EMT by functioning as a sponge for miR‐1264 and QKI. Therefore, we confirmed that circDDX21 repressed EMT in TNBC cells. Subsequent rescue assays also confirmed that miR‐1264 and QKI also participated in the regulation of circDDX21 and EMT. Actually, QKI has been reported to contribute to the EMT in various cancers. During the EMT process, QKI‐5 directly binds to and regulates alternative splicing targets, thus exerting multiple effects like increasing cell migration and invasion and inhibiting tumour growth without significantly altering mRNA levels.^10^

In conclusion, our research identified a new circRNA, circDDX21, as a prognostic indicator for TNBC. CircDDX21 functions as a sponge for miR‐1264 and regulates QKI expression, thus repressing EMT in TNBC (Figure S11). Therefore, our research provides a potential diagnostic biomarker and therapeutic target for patients with TNBC.

## 1 METHODS

### 1.1 Patients and specimens

Sixty‐six TNBC specimens and paired non‐tumour breast specimens were collected from July 2007 to July 2017. Patients with the following criteria were excluded from participation: had received adjuvant chemotherapy or radiotherapy prior to surgery; and had additional cancer diagnoses. All patients were classified according to the 7th edition of the TNM staging system. Postoperative adjuvant therapies were administered according to standard schedules and doses. All participating patients provided written informed consent. This study was approved by the ethics committee of the authors’ hospital. The clinical data for all patients is shown in Table S1.

### 1.2 Cell culture and infection

The human TNBC cell lines MCF7, T47D, SK‐BR3, BT474, MDA‐MB‐231, SUM1315 and BT549; the human normal mammary gland cell line MCF10A and the mouse TNBC cell lines 4T1 and E0771 were all purchased from the Shanghai Cell Bank, Chinese Academy of Sciences. The cells were cultured in DMEM high glucose medium supplemented with 10% FBS (Sigma‐Aldrich, USA) and a 1% penicillin‐streptomycin solution (Beyotime, China) at 37°C in a 5% CO_2_ atmosphere.

The shRNA and overexpression vectors for human circDDX21 were synthesised by Bioegene Co., Ltd. (Shanghai, China). In accordance with the instructions of the product manual, Lipofectamine 3000 (Invitrogen, Inc.) was used to co‐transfect the target plasmid or the scrambled vector, psPAX2 or PMG.2G, respectively, into HEK293T tool cells to obtain a circDDX21 knockdown lentivirus or overexpression lentivirus. Then, the lentivirus (multiplicity of infection, MOI = 10) was used to infect TNBC cells. Seventy‐two hours after infection, the cells were further screened by treating them with puromycin (2 μg/ml, 72 h).

### 1.3 RNA extraction, reverse transcription and quantitative PCR (RT‐qPCR)

Total RNA was extracted using TRIzol reagent (Invitrogen). Genomic DNA (gDNA) was isolated with FastPure DNA Isolation (Vazyme, China). For the analysis of circRNAs and mRNAs, reverse transcription was performed using the PrimeScript™ RT reagent kit (Takara Bio, Inc., Otsu, Japan) with random primers. For the analysis of miRNAs, reverse transcription was performed using the PrimeScript RT Reagent Kit (Takara, Japan) with specific stem‐loop primers. The expression of RNAs was assessed using real‐time quantitative PCR in triplicate with a SYBR Premix Ex Taq™ kit (Takara Bio) and ABI 7900HT Real‐Time PCR system (Applied Biosystems Life Technologies, Foster City, CA, USA). The primers used to amplify circRNAs are listed in Table S2, and the primers used to amplify miRNAs are listed in Table S3. Comparative cycle threshold values (2^−ΔΔCt^) were adopted to analyse the final results.

### 1.4 Fluorescence in situ hybridisation (FISH) assay

Tissues or cells on slides were fixed with 4% paraformaldehyde, followed by permeabilisation with 0.5% Triton X‐100. Then, the Cy3‐labelled circDDX21 (5′‐Cy3‐GGTCACTCCTCGGCCTTTCTTGTATGCCAC‐Cy3‐3′) probe and FAM‐labelled miR‐1264 probes (5′‐FAM‐AACAGGTGCTCAAATAAGACTTG‐FAM‐3′) were diluted to 1 μM and incubated with slides at 42°C overnight. Finally, slides were further stained with DAPI for 5 min at room temperature, and images were acquired with a Leica confocal microscope (Leica Microsystems, Germany).

### 1.5 Proliferation and clone formation assays

For the proliferation assay, 3 × 10^3^ cells suspended in 100 μl of RPMI‐1640 medium were seeded in 96‐well plates. Cell proliferation was assessed using CCK‐8 (Dojindo Molecular Technologies, Japan). Ten microliters of the CCK‐8 solution were added to each well of the plate. Finally, we measured the absorbance at 450 nm after a 2 h incubation.

For the clone formation assay, 500 cells were seeded into 6‐well plates and incubated at 37°C. The clone size was observed daily under a microscope until the number of cells in the majority of clones was >50. Then, the medium was removed, and the cells were stained with 0.2% crystal violet for 30 min. Cells were washed three times with PBS and then photographed, and the clones were counted. The ratio of clone formation was calculated with the following equation: Ratio of clone formation (%) = clone number/500 × 100.

### 1.6 Wound healing assay

A total of 5 × 10^5^ cells were seeded into 6‐well culture plates, and a sterile micropipette tip was used to scratch a straight line in the confluent monolayers. The cells were incubated to allow them to cover the wound for 24 h, and pictures of the same wound were captured under a microscope. The wound areas were analysed using ImageJ software.

### 1.7 Transwell assays

Cell migration and invasion were analysed with Transwell plates (24‐well insert, 8 μm pore size; BD Biosciences, Bedford, MA, USA). The filters (Corning Inc., USA) were coated with (invasion assay) or without (migration assay) 55 μl of Matrigel (1:8 dilution; BD Biosciences). Then, 10^4^ cells were suspended in 100 μl of serum‐free medium and seeded in the upper chamber. Next, 600 μl of 90% medium supplemented with 10% FBS were added to the bottom chamber. After an incubation for 24 h, the chambers were fixed with 4% paraformaldehyde for 30 min and then stained with 0.1% crystal violet for 30 min. Finally, we used a microscope to count the number of invading cells in the bottom of the chamber.

### 1.8 Western blot

Total proteins were extracted from each group of cells using RIPA buffer supplemented with 1% phenylmethylsulfonyl fluoride (PMSF). The protein concentration in each group was quantified using a BCA protein assay kit (Pierce, 23227). Generally, an equal amount of 20 μg of protein was separated on 10% SDS polyacrylamide gels and then transferred onto polyvinylidene difluoride (PVDF) membranes. Subsequently, the membranes were blocked with a 5% bovine serum albumin (BSA) solution for 1 h at room temperature. Then, the membranes were incubated with primary antibodies against GAPDH (1:1000, Epizyme Biotech, LF206), E‐cadherin (1:1000, Cell Signaling Technology, #3195), N‐cadherin (1:1000, Cell Signaling Technology, #13116), Vimentin (1:1000, Cell Signaling Technology, #5741), and QKI (1:1000, Absin Bioscience Inc., abs117610), AKT (1:1000, Cell Signaling Technology, #9272), p‐AKT (1:1000, Cell Signaling Technology, #4060), ERK1/2 (1:1000, Cell Signaling Technology, #4695), p‐ERK1/2 (1:1000, Cell Signaling Technology, #4370), STAT3 (1:1000, Cell Signaling Technology, #12640), p‐STAT3 (1:1000, Cell Signaling Technology, #9145), p38 MAPK (1:1000, Proteintech, 14064‐1‐AP), p‐p38 MAPK (1:1000, Proteintech, 28796‐1‐AP) at 4°C overnight. Next, the membranes were incubated with a goat anti‐rabbit secondary antibody (1:3000) and detected using an enhanced chemiluminescence (ECL) detection system (Thermo Scientific, USA).

### 1.9 Immunofluorescence staining

Cells growing onto coverslips were fixed with 4% paraformaldehyde for 15 min and permeabilised with 0.2% Triton X‐100 for another 20 min. Then, the coverslips were incubated with the E‐cadherin antibody (1:200, Cell Signaling Technology, #3195), N‐cadherin antibody (1:200, Cell Signaling Technology, #13116) and Vimentin antibody (1:200, Cell Signaling Technology, #5741) at 4°C overnight. Subsequently, the cells were incubated with Alexa Fluor 596‐conjugated goat anti‐rabbit IgG antibodies (1:200; Proteintech Group, Shanghai, China) at room temperature for 1 h at 4°C in the dark. DAPI was applied to counterstain cell nuclei for 20 min. Images were photographed under a laser scanning confocal microscope.

### 1.10 Immunohistochemical (IHC) staining

Sectioned tumour tissues from the homograft model were embedded in paraffin, rehydrated and blocked by an incubation with goat serum. Sections were incubated with an E‐cadherin antibody (1:400, Cell Signaling Technology, #3195), N‐cadherin antibody (1:400, Cell Signaling Technology, #13116) and Vimentin antibody (1:400, Cell Signaling Technology, #5741) at 4°C overnight, followed by an incubation with an HRP‐conjugated goat anti‐rabbit (Envision‐AP, Dako, Denmark, EU) secondary antibody at 25°C for 1 h. The sections were treated with the Metal Enhanced DAB Substrate Kit (Dako, Denmark, EU) and stained with haematoxylin (Beyotime, China). Finally, the density of target proteins was measured by calculating integrated optical density (IOD) using ImageJ software.

### 1.11 Luciferase reporter assay

This assay was performed according to the instructions of the Dual‐Glo Luciferase Assay System (Promega Corp., Madison, WI, USA). Wild‐type or mutated circDDX21 or QKI containing the predicted miR‐1264 binding sites was inserted into the dual‐luciferase reporter vector psi‐CHECK‐2. Thirty‐six hours after transfection, the cells were lysed with passive lysis buffer. Firefly luciferase (F‐luc) and Renilla luciferase (R‐luc) activities in the lysates were detected. Transcriptional activity was presented as F‐luc/R‐luc, and the translation efficiency was further normalised to F‐luc mRNA expression.

### 1.12 RNA pull‐down assay

The biotin‐labelled circDDX21 and a random oligonucleotide probe were acquired from RiboBio (Guangzhou, China) and incubated with streptavidin Dynabeads (Invitrogen, USA) at room temperature for 2 h. Then, the lysates of TNBC cells were incubated with both probes and streptavidin beads at 4°C overnight. Subsequently, the lysates were washed three times and eluted from the beads. Next, the enrichment of miRNAs in the precipitated complexes was evaluated using RT‐qPCR as described above.

### 1.13 Subcutaneous homograft model and lung metastasis model

For subcutaneous homografts, 5‐week‐old Balb/c mice were provided by the Beijing Vital River Laboratory Animal Technology Co. Ltd. All mice (*n* = 6 per group) were equally and randomly divided into the Sh‐NC, Sh‐circDDX21, vector and circDDX21 groups. A total of 3 × 10^6^ 4T1 or E0771 cells suspended in 100 μl of PBS were injected subcutaneously at the axilla of each nude mouse. After 1 week, the long (*L*) and short (*S*) diameters of the tumours were measured with Vernier calliper every 3 days (tumour volume = *L* × *S*2/2). The growth curve of subcutaneous tumours was drawn based on the measured tumour volume. All mice were euthanised 3 weeks after the injection of CRC cells, and subcutaneous tumours were removed completely. The tumours were weighed and prepared into paraffin section.

The groups for the lung metastasis model were the same as those for the subcutaneous homograft model described above. Mice were injected with 1 × 10^6^ TNBC cells via tail vein. All mice were euthanised 1 week after the injection of TNBC cells. The lungs were removed completely and stained with haematoxylin‐eosin (HE). All tissues were observed and photographed under a microscope.

### 1.14 Statistical analysis

All analyses were performed using SPSS software (version 22.0, IBM Corp., Armonk, NY, USA). All statistical tests were two‐sided, and a *p* value < .05 was considered statistically significant. Continuous variables that conformed to the normal distribution were compared with independent *t*‐test between groups, while continuous variables with a skewed distribution were compared with the Mann–Whitney *U* test. The relationship between hub genes and overall survival was analysed by constructing a Kaplan–Meier curve, which was evaluated by log‐rank test. A univariate regression model was used to analyse the effects of individual variables on survival, and a multivariate Cox regression model was used to confirm independent influencing factors associated with survival.

## CONFLICT OF INTEREST

All the authors declared that no conflicts of interest were involved in this study.

## CONSENT FOR PUBLICATION

All procedures involving human participants were performed in accordance with the ethics committee of the authors' institution and with the 1964 Declaration of Helsinki and its later amendments or comparable ethical standards. All patients provided their written informed consent.

## Supporting information


**FIGURE S1**. The circRNA‐forming exonic sequences of circDDX21 were validated by Sanger sequencing and agarose gel electrophoresis

**FIGURE S2**. CircDDX21 regulates 4T1 cell proliferation and invasion in vitro. (A) Overexpression and knockdown of circDDX21 in 4T1 cells validated by RT‐qPCR. (B) The proliferation of 4T1 cells after overexpression and knockdown of circDDX21. (C and D) The migration of 4T1 cells was determined by wound healing assays. (E and F) The migration and invasion of 4T1 cells were determined by Transwell assays (^*^ *p*  < .05, ^**^ *p*  < .01, ^***^ *p*  < .001)

**FIGURE S3**. CircDDX21 regulates cell proliferation and invasion in vitro. (A) Representative images of wound healing assays of SUM 1315 and MDA‐MB 231 cells. (B) Representative images of Transwell migration and invasion assays of SUM 1315 and MDA‐MB 231 cells. (C) Representative images of clone formation of SUM 1315 and MDA‐MB 231 cells.

**FIGURE S4**. CircDDX21 regulates E0771 cell growth, invasion and metastasis in vivo. (A and B) The homografts and growth curve derived from the circDDX21‐overexpressing and circDDX21‐inhibited E0771 cells (*n* = 6 per group). (C–E) CircDDX21 regulated lung metastasis of E0771 cells (^**^ *p*  < .01, ^***^ *p*  < .001)

**FIGURE S5**. CircDDX21 acts as a sponge for miR‐1264. (A) Representative images of wound healing assays of the circDDX21 knockdown cells transfected with the miR‐1264 inhibitor. (B) Representative images of Transwell migration and invasion of the circDDX21 knockdown cells transfected with the miR‐1264 inhibitor. (C) Representative images of clone formation of the circDDX21 knockdown cells transfected with the miR‐1264 inhibitor.

**FIGURE S6**. QKI is a direct target of miR‐1264. (A) Representative images of wound healing assays of the miR‐1264‐inhibited cells transfected with QKI. (B) Representative images of the Transwell migration and invasion of the circDDX21 knockdown cells transfected with the miR‐1264 inhibitor. (C) Representative images of clone formation of the miR‐1264‐inhibited cells transfected with QKI.

**FIGURE S7**. (A–D) The expression of E‐cadherin, N‐cadherin and Vimentin in the circDDX21 knockdown and circDDX21‐overexpressing cells was assessed by Western blotting. (E–H) The expression of E‐cadherin, N‐cadherin and Vimentin in the circDDX21 knockdown and circDDX21‐overexpressing cells was determined by IF (^*^ *p*  < .05, ^**^ *p*  < .01, ^***^ *p*  < .001)

**FIGURE S8**. Inhibition of miR‐1264 rescued the changes in E‐cadherin, N‐cadherin and Vimentin in the circDDX21 knockdown cells. (A–D) Western blotting analysis. (E–H) IF assay (^*^ *p*  < .05, ^**^ *p*  < .01, ^***^ *p*  < .001)

**FIGURE S9**. Overexpression of QKI rescued the changes in E‐cadherin, N‐cadherin and Vimentin in the miR‐1264‐overexpressing cells. (A–D) Western blotting analysis. (E–H) IF assay (^*^ *p*  < .05, ^**^ *p*  < .01, ^***^ *p*  < .001)

**FIGURE S10**. QKI regulates cell proliferation via inhibition of the p38 MAPK signalling pathway. (A and B) The expression levels of AKT, p‐AKT, STAT3, p‐STAT3, ERK1/2, p‐ERK1/2, p38 MAPK and p‐p38 MAPK in the QKI‐overexpressing cells and the control cells. (C and D) A p‐p38 MAPK inhibitor (SB203580) restored the level of p‐p38 MAPK in the QKI‐overexpressing SUM 1315 and MDA‐MB‐231 cells. (E–H) Cell proliferation and colony formation abilities were detected in the QKI‐overexpressing SUM 1315 and MDA‐MB‐231 cells treated with a p‐p38 MAPK inhibitor.

**FIGURE S11**. The role of the circDDX21/miR‐1264/QKI axis in TNBC

**TABLE S1** Association between circDDX21 and the clinicopathological characteristic of TNBC
**TABLE S2** The primer sequences of circRNAs
**TABLE S3** The primer sequence of relevant miRNAs
