# Supplementary figures and images for: CircRNA DDX21 acts as a prognostic factor and sponge of miR‐1264/QKI axis to weaken the progression of triple‐negative breast cancer
Source: Clin Transl Med. 2022 May 6;12(5):e768. doi: 10.1002/ctm2.768 (PMC9076009; doi:10.1002/ctm2.768)

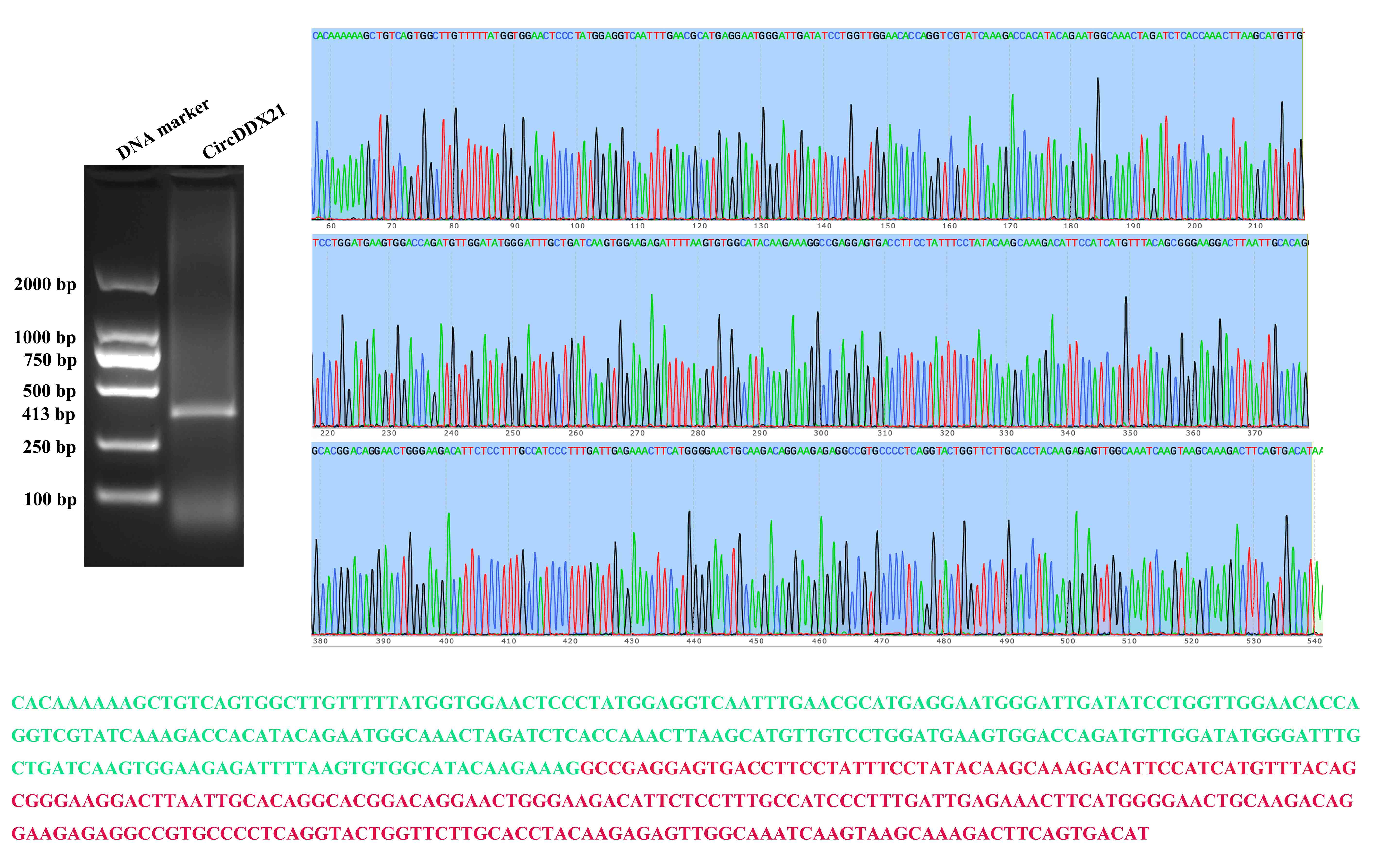

Supplement: Supplementary file 1 — FIGURE S1. The circRNA‐forming exonic sequences of circDDX21 were validated by Sanger sequencing and agarose gel electrophoresis [file CTM2-12-e768-s012.jpg]

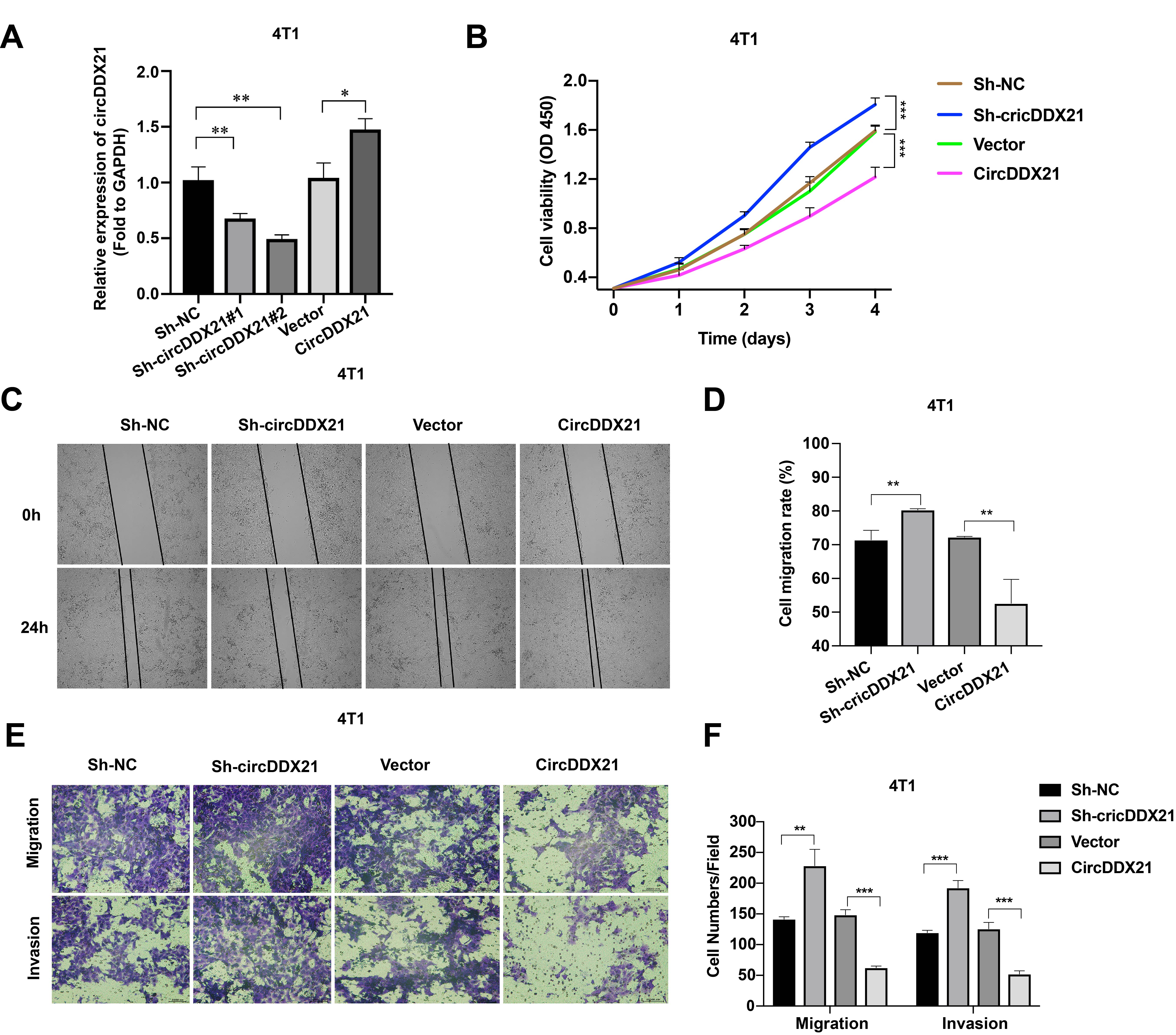

Supplement: Supplementary file 2 — FIGURE S2. CircDDX21 regulates 4T1 cell proliferation and invasion in vitro. (A) Overexpression and knockdown of circDDX21 in 4T1 cells validated by RT‐qPCR. (B) The proliferation of 4T1 cells after overexpression and knockdown of circDDX21. (C and D) The migration of 4T1 cells was determined by wound healing assays. (E and F) The migration and invasion of 4T1 cells were determined by Transwell assays (* p < .05, ** p < .01, *** p < .001) [file CTM2-12-e768-s011.jpg]

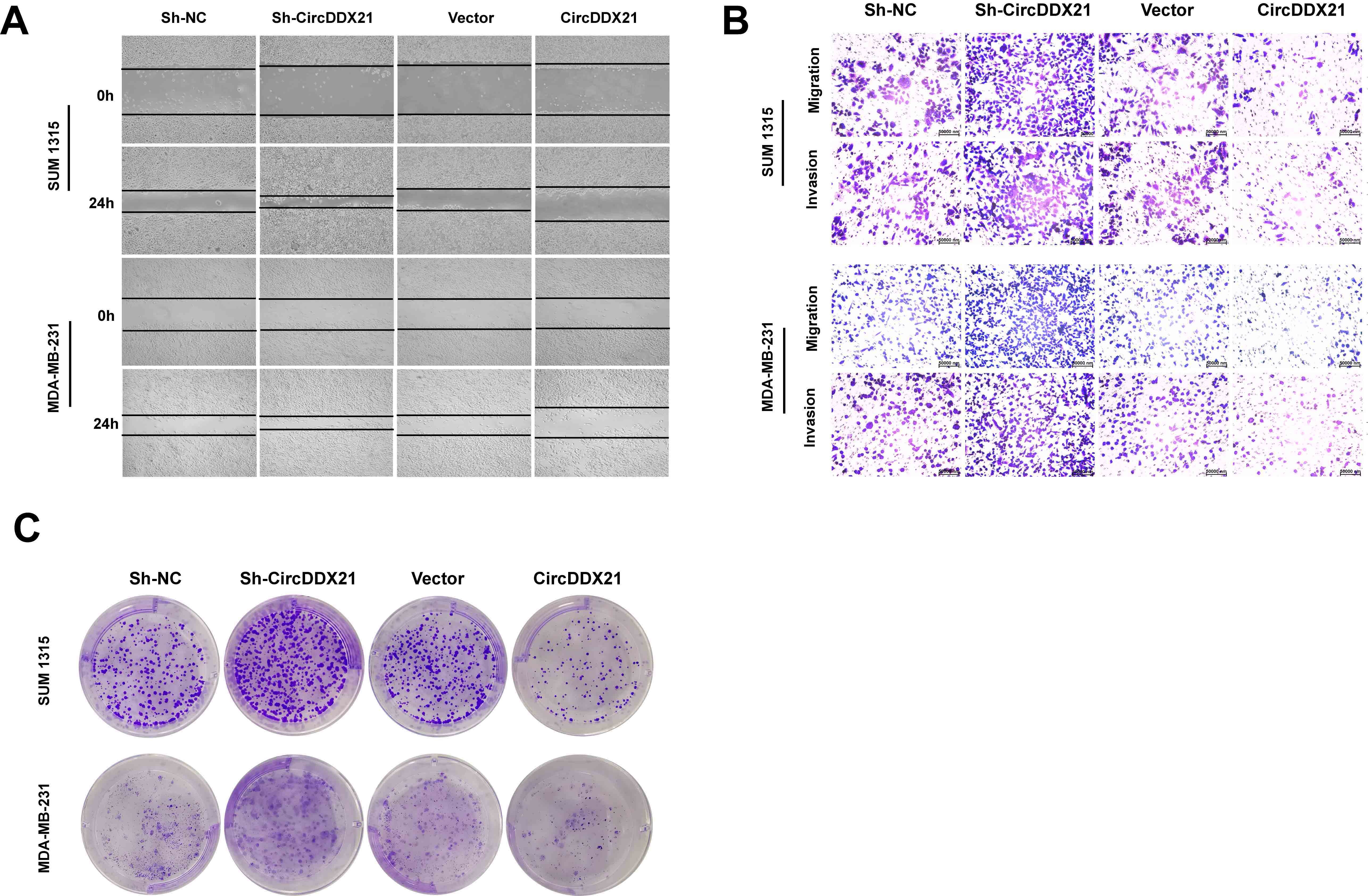

Supplement: Supplementary file 3 — FIGURE S3. CircDDX21 regulates cell proliferation and invasion in vitro. (A) Representative images of wound healing assays of SUM 1315 and MDA‐MB 231 cells. (B) Representative images of Transwell migration and invasion assays of SUM 1315 and MDA‐MB 231 cells. (C) Representative images of clone formation of SUM 1315 and MDA‐MB 231 cells. [file CTM2-12-e768-s006.jpg]

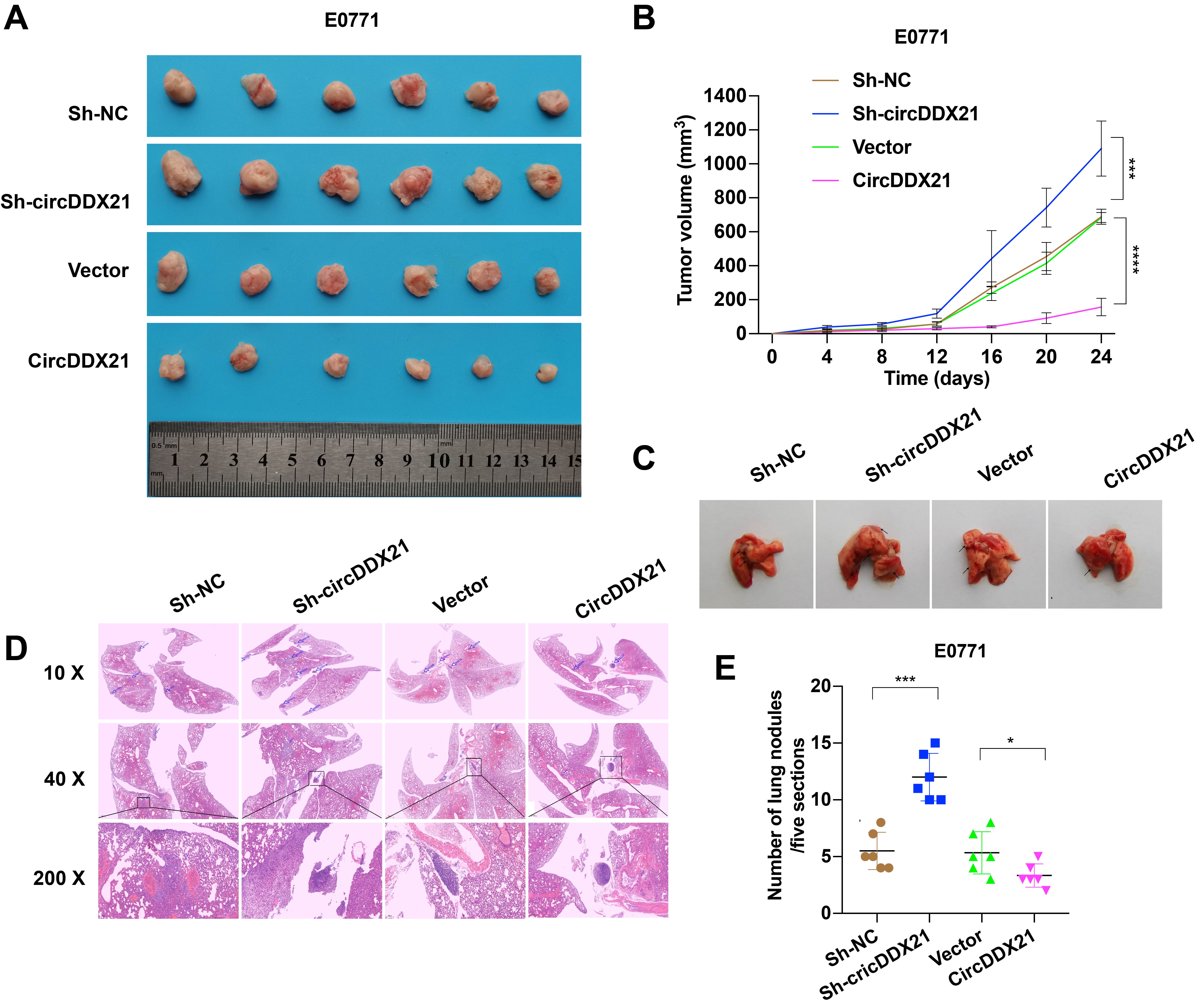

Supplement: Supplementary file 4 — FIGURE S4. CircDDX21 regulates E0771 cell growth, invasion and metastasis in vivo. (A and B) The homografts and growth curve derived from the circDDX21‐overexpressing and circDDX21‐inhibited E0771 cells (n = 6 per group). (C–E) CircDDX21 regulated lung metastasis of E0771 cells (** p < .01, *** p < .001) [file CTM2-12-e768-s002.jpg]

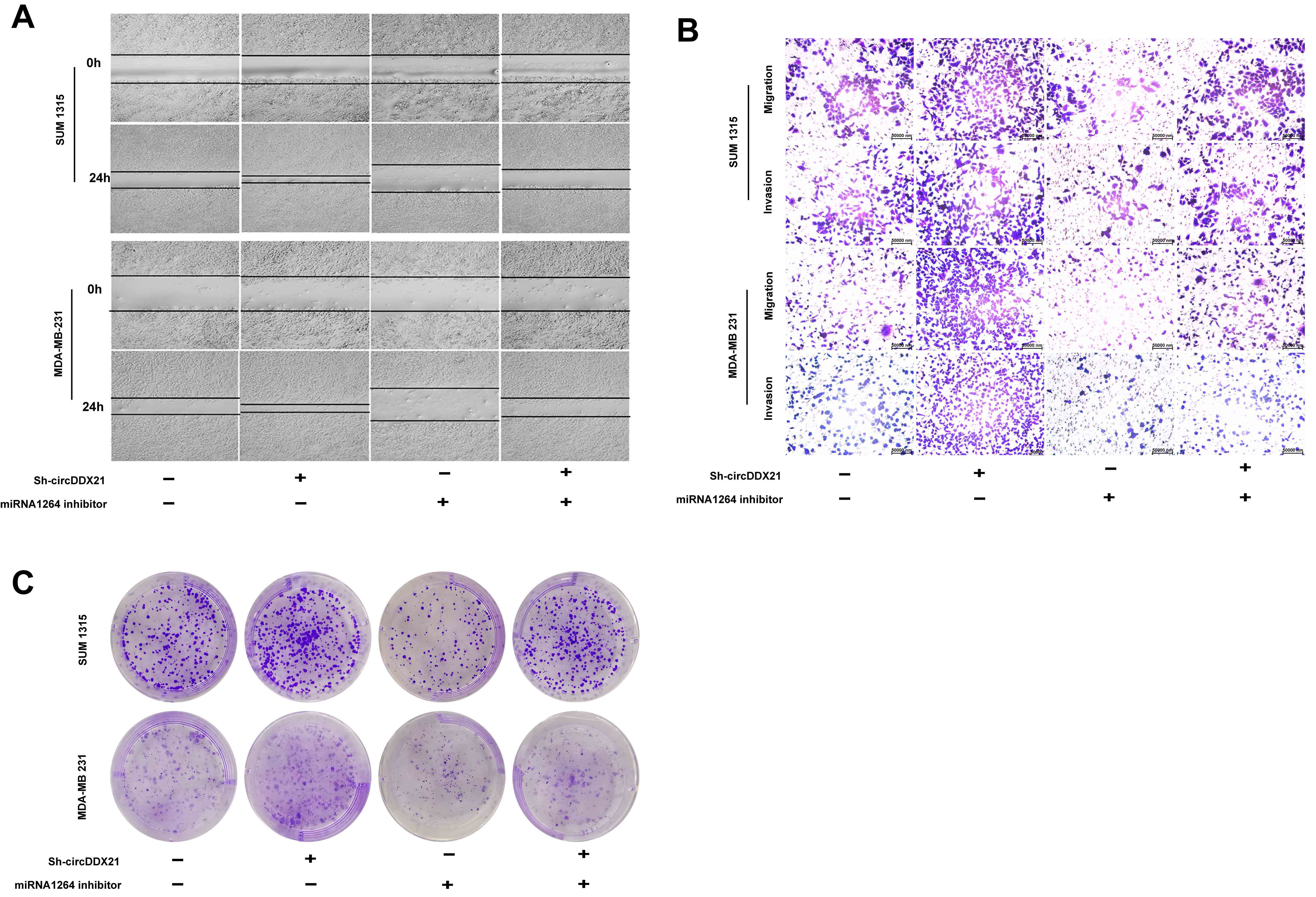

Supplement: Supplementary file 5 — FIGURE S5. CircDDX21 acts as a sponge for miR‐1264. (A) Representative images of wound healing assays of the circDDX21 knockdown cells transfected with the miR‐1264 inhibitor. (B) Representative images of Transwell migration and invasion of the circDDX21 knockdown cells transfected with the miR‐1264 inhibitor. (C) Representative images of clone formation of the circDDX21 knockdown cells transfected with the miR‐1264 inhibitor. [file CTM2-12-e768-s005.jpg]

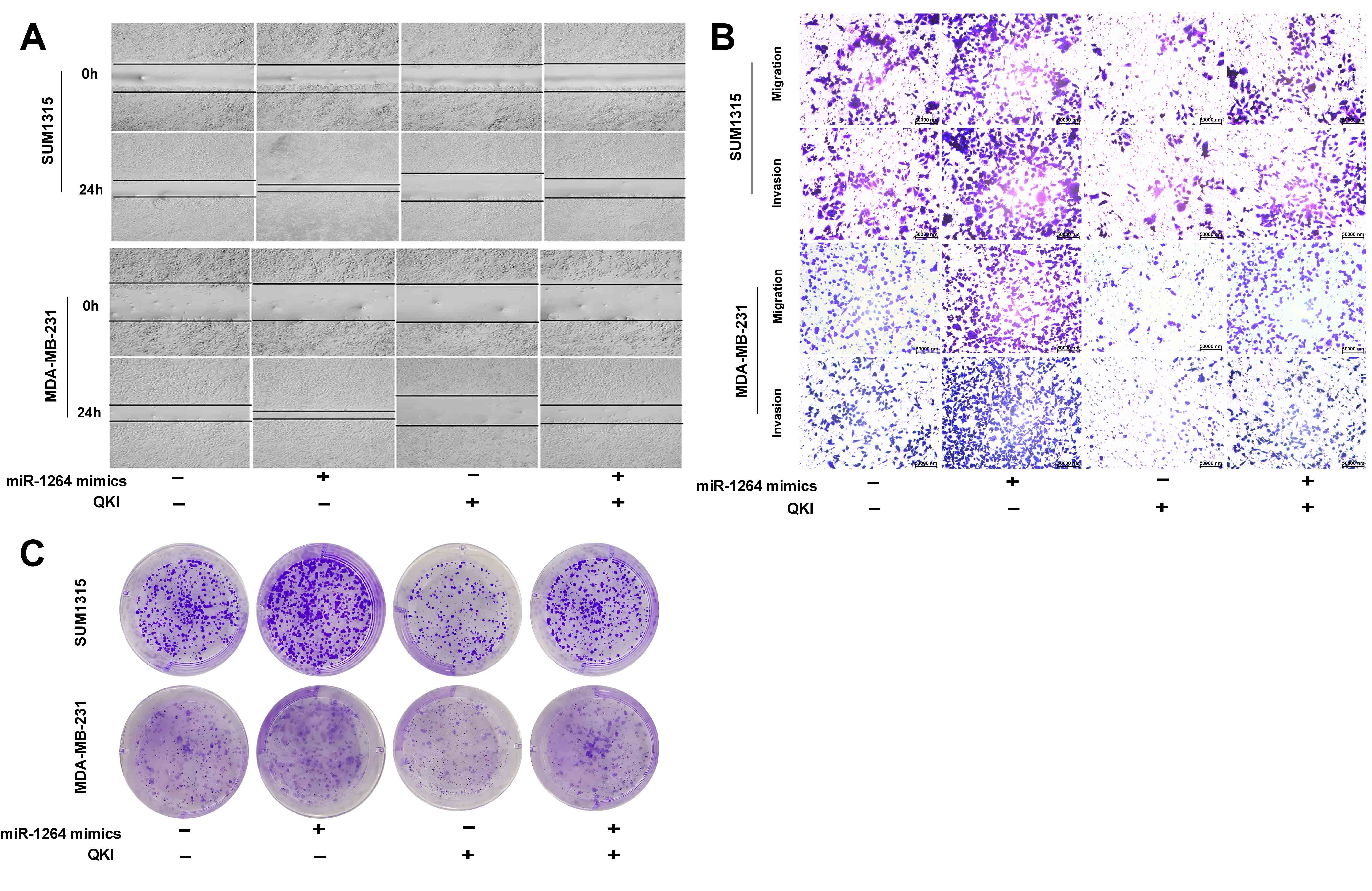

Supplement: Supplementary file 6 — FIGURE S6. QKI is a direct target of miR‐1264. (A) Representative images of wound healing assays of the miR‐1264‐inhibited cells transfected with QKI. (B) Representative images of the Transwell migration and invasion of the circDDX21 knockdown cells transfected with the miR‐1264 inhibitor. (C) Representative images of clone formation of the miR‐1264‐inhibited cells transfected with QKI. [file CTM2-12-e768-s003.jpg]

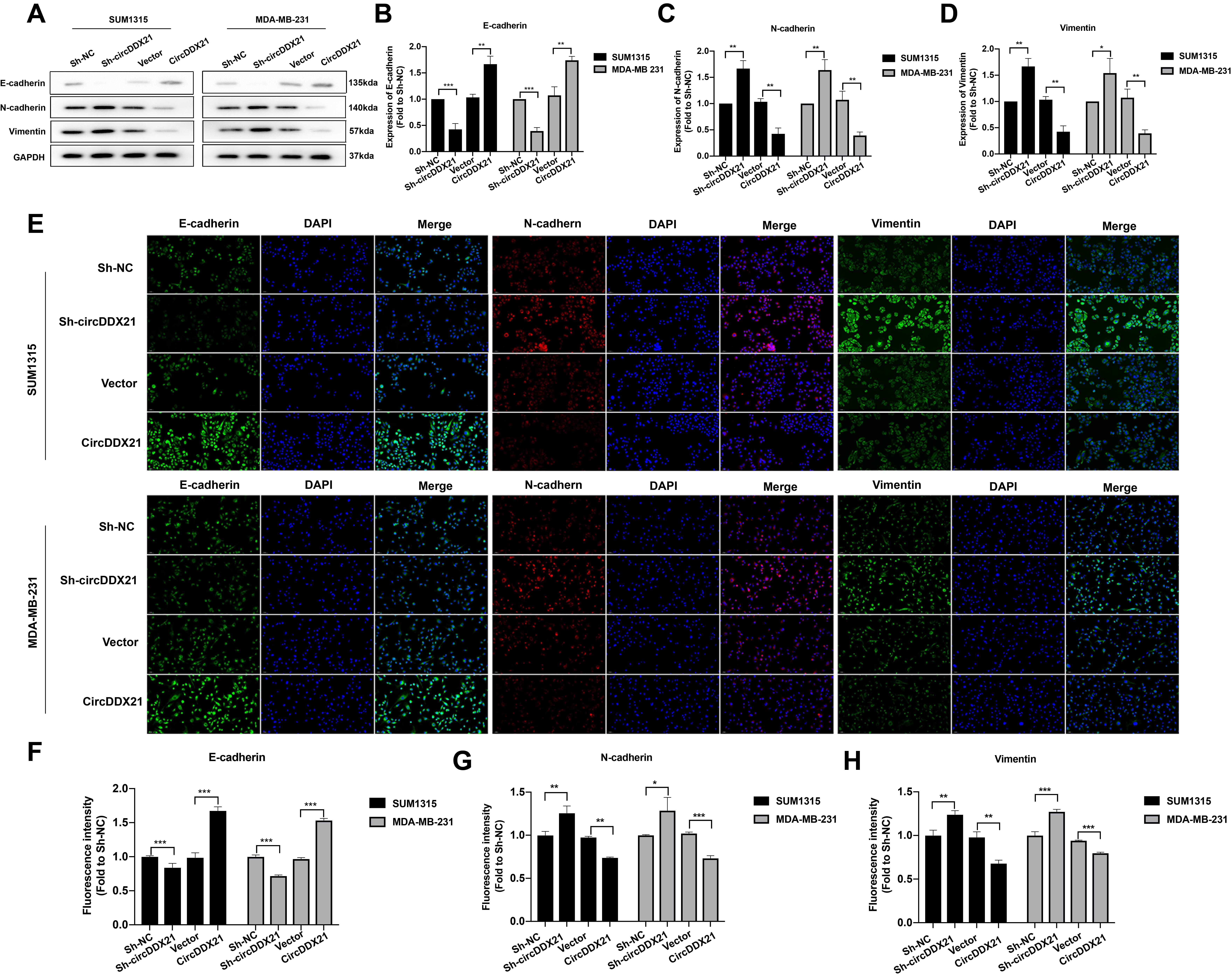

Supplement: Supplementary file 7 — FIGURE S7. (A–D) The expression of E‐cadherin, N‐cadherin and Vimentin in the circDDX21 knockdown and circDDX21‐overexpressing cells was assessed by Western blotting. (E–H) The expression of E‐cadherin, N‐cadherin and Vimentin in the circDDX21 knockdown and circDDX21‐overexpressing cells was determined by IF (* p < .05, ** p < .01, *** p < .001) [file CTM2-12-e768-s009.jpg]

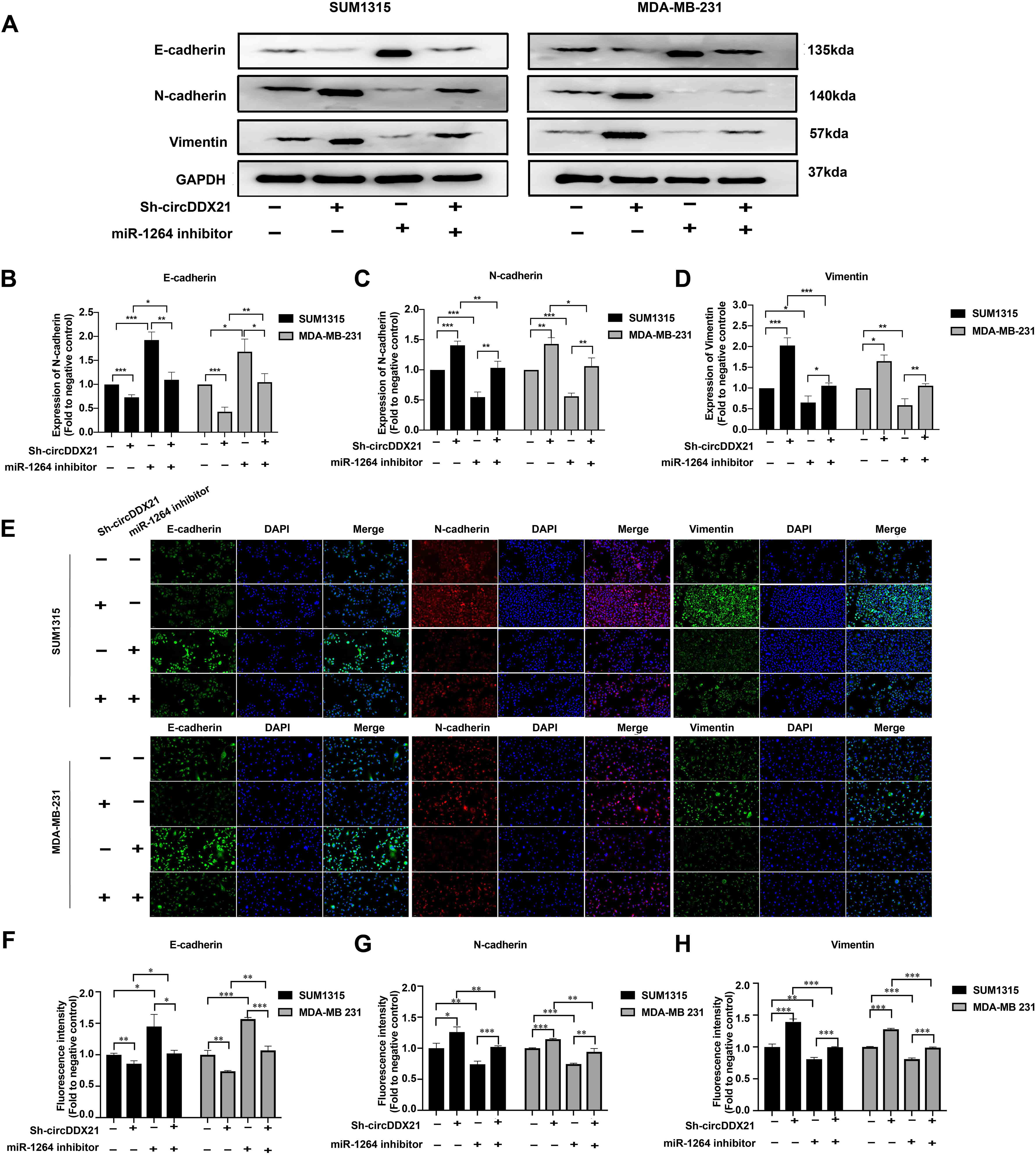

Supplement: Supplementary file 8 — FIGURE S8. Inhibition of miR‐1264 rescued the changes in E‐cadherin, N‐cadherin and Vimentin in the circDDX21 knockdown cells. (A–D) Western blotting analysis. (E–H) IF assay (* p < .05, ** p < .01, *** p < .001) [file CTM2-12-e768-s001.jpg]

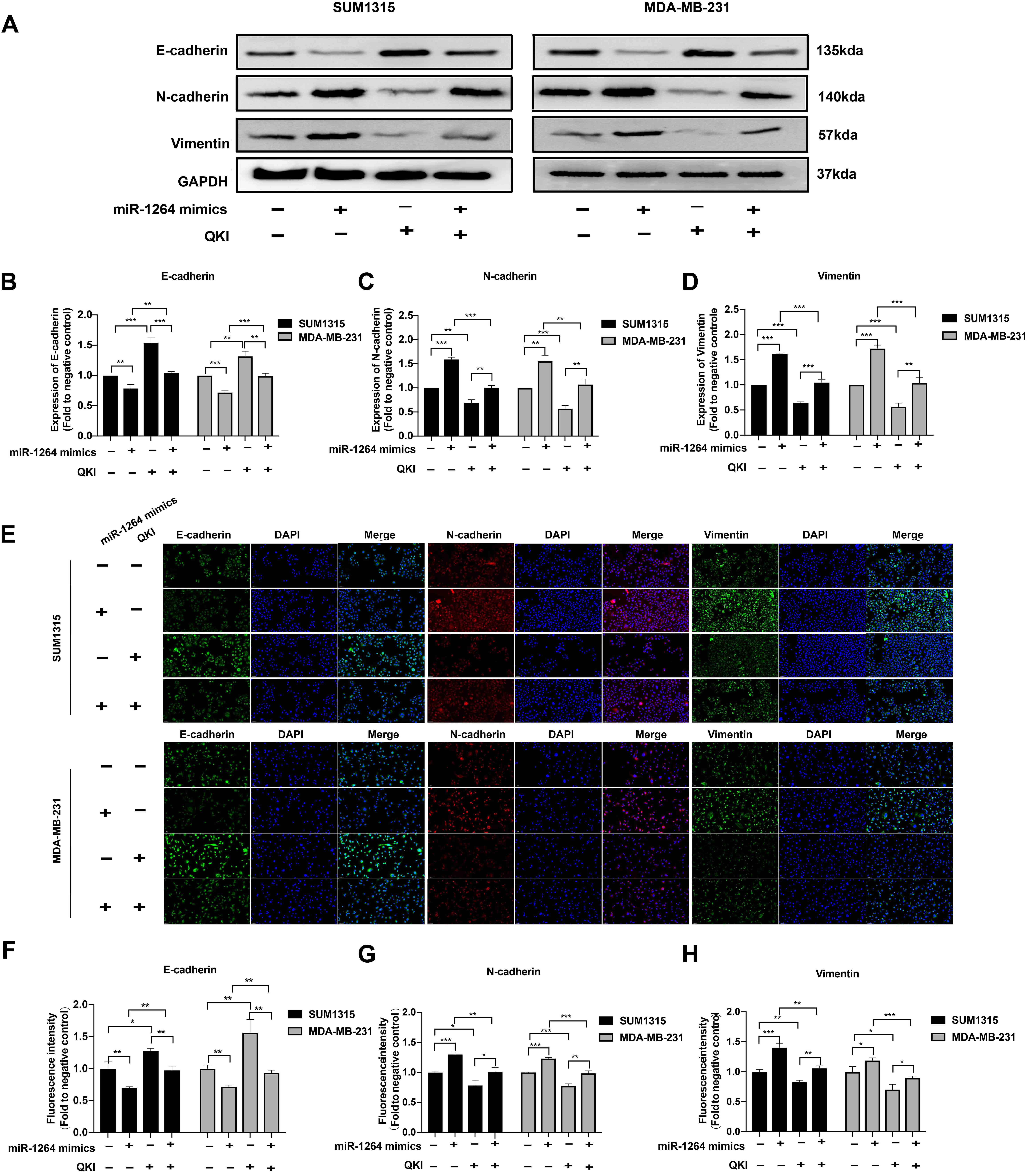

Supplement: Supplementary file 9 — FIGURE S9. Overexpression of QKI rescued the changes in E‐cadherin, N‐cadherin and Vimentin in the miR‐1264‐overexpressing cells. (A–D) Western blotting analysis. (E–H) IF assay (* p < .05, ** p < .01, *** p < .001) [file CTM2-12-e768-s007.jpg]

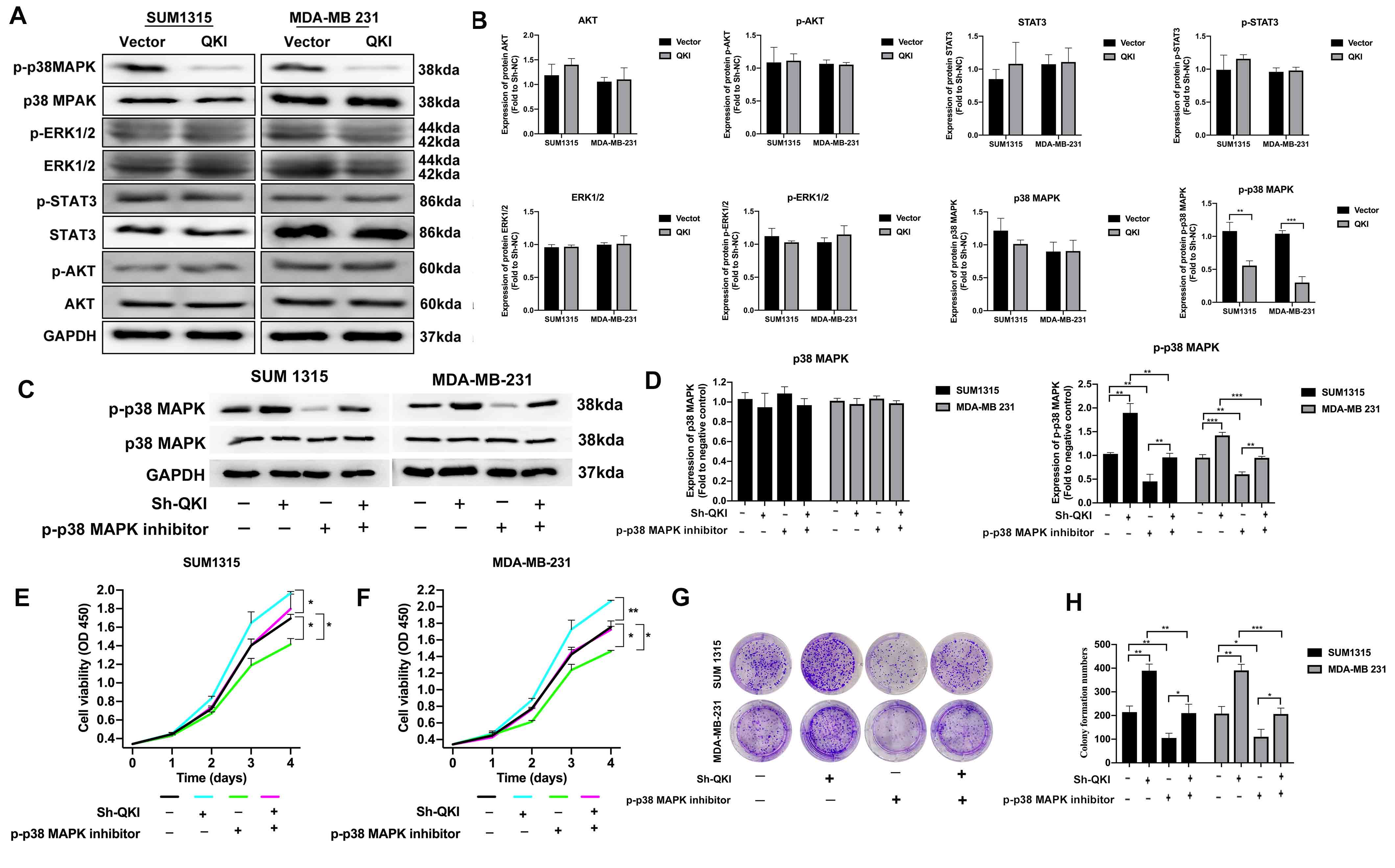

Supplement: Supplementary file 10 — FIGURE S10. QKI regulates cell proliferation via inhibition of the p38 MAPK signalling pathway. (A and B) The expression levels of AKT, p‐AKT, STAT3, p‐STAT3, ERK1/2, p‐ERK1/2, p38 MAPK and p‐p38 MAPK in the QKI‐overexpressing cells and the control cells. (C and D) A p‐p38 MAPK inhibitor (SB203580) restored the level of p‐p38 MAPK in the QKI‐overexpressing SUM 1315 and MDA‐MB‐231 cells. (E–H) Cell proliferation and colony formation abilities were detected in the QKI‐overexpressing SUM 1315 and MDA‐MB‐231 cells treated with a p‐p38 MAPK inhibitor. [file CTM2-12-e768-s008.jpg]

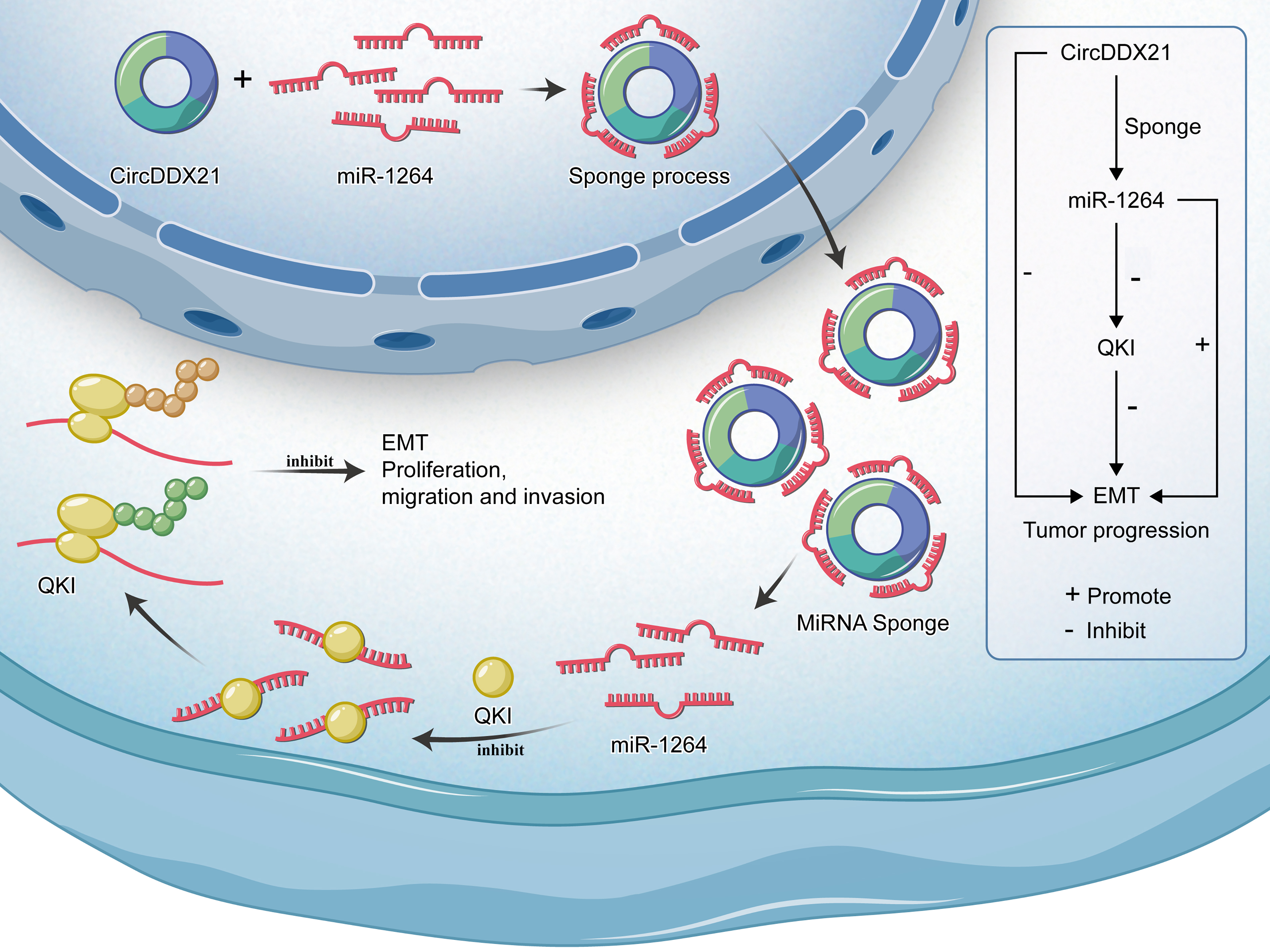

Supplement: Supplementary file 11 — FIGURE S11. The role of the circDDX21/miR‐1264/QKI axis in TNBC [file CTM2-12-e768-s004.jpg]
