# Supplementary material for: CircRNA DDX21 acts as a prognostic factor and sponge of miR‐1264/QKI axis to weaken the progression of triple‐negative breast cancer
Source: Clin Transl Med. 2022 May 6;12(5):e768. doi: 10.1002/ctm2.768 (PMC9076009; doi:10.1002/ctm2.768)
Supplement: Supplementary file 12 — TABLE S1 Association between circDDX21 and the clinicopathological characteristic of TNBC TABLE S2 The primer sequences of circRNAs TABLE S3 The primer sequence of relevant miRNAs [file CTM2-12-e768-s010.docx]

**Table S1 Association between circDDX21 and the clinicopathological characteristic of TNBC**

| Variables | Cases(n=66) | | CircDDX21 | | *P* value |
| --- | --- | --- | --- | --- | --- |
|  |  |  | **Low(n=40)** | **High(n=26)** |  |
| Age (year) |  | |  |  | 0.744 |
| <50 | 39 | | 23 | 16 |  |
| ≥50 | 27 | | 17 | 10 |  |
| Tumor Grade |  | |  |  | **0.031** |
| I-II | 25 | | 11 | 14 |  |
| III | 41 | | 29 | 12 |  |
| T stage |  | |  |  |  |
| T<2cm | 7 | | 3 | 4 | 0.309 |
| T>=2cm | 59 | | 37 | 22 |  |
| Lymph node metastasis | |  |  |  | **<0.001** |
| N0 | 25 | | 11 | 14 |  |
| N+ | 41 | | 29 | 12 |  |
| TNM stage |  | |  |  | **<0.001** |
| I | 4 | | 0 | 4 |  |
| II | 28 | | 12 | 16 |  |
| III | 27 | | 21 | 6 |  |
| IV | 7 | | 7 | 0 |  |

**Table S2 The primer sequences of circRNAs.**

| Genes | Forward primer | Reverse primer |
| --- | --- | --- |
| hsa_circ_0005699 | CATTCTGGTCAGGTGGCCTT | AGCTGGTTCTGGACCGTATC |
| hsa_circ_0000517 | GTGAGTTCCCAGAGAACGGG | CAGGGAGAGCCCTGTTAGG |
| hsa_circ_0000520 | AAGGTCTGAGACTAGGGCCA | GATGAGCTTCCCTCCCCGAA |
| hsa_circ_0000519 | TAACAGGGCTCTCCCTGAGC | TTCCCAAGGGACATGGGAGT |
| hsa_circ_0000516 | GCTCAGGGAGAGCCCTGTTA | GGTGAGTTCCCAGAGAACGG |
| hsa_circ_0000375 | CGACTGTGACAGCCTGACTT | TGGATGGGACCTCTCCCTG |
| hsa_circ_0008523 | TGGCATACAAGAAAGGCCGA | TGTCCGTGCCTGTGCAATTA |
| hsa_circ_0028899 | AAAGGGGTGTTGGTGGCTTT | TGGCTACAAATGCGCTCCTA |
| hsa_circ_0000376 | CACTCATCGAAGACTGGCGT | CGTATGGAATGGACCTGGACA |

**Table S3. The primer sequence of relevant miRNAs.**

| Genes | Forward primer | Reverse primer |
| --- | --- | --- |
| hsa_miR_1236 | ACACTCCAGCTGGGTGAGTGACAGGGGAAATG | CTCAACTGGTGTCGTGGAGTCGGCAATTCAGTTGAGTCCCCA |
| hsa_miR_1264 | ACACTCCAGCTGGGCAAGTCTTATTTGAGCAC | CTCAACTGGTGTCGTGGAGTCGGCAATTCAGTTGAGAACAGG |
| hsa_miR_192 | ACACTCCAGCTGGGCTGACCTATGAATTGAC | CTCAACTGGTGTCGTGGAGTCGGCAATTCAGTTGAGGGCTGT |
| hsa_miR_215 | ACACTCCAGCTGGGATGACCTATGAATTGAC | CTCAACTGGTGTCGTGGAGTCGGCAATTCAGTTGAGGTCTGT |
| hsa_miR_383 | ACACTCCAGCTGGGAGATCAGAAGGTGATTG | CTCAACTGGTGTCGTGGAGTCGGCAATTCAGTTGAGAGCCAC |
| hsa_miR_ 431 | ACACTCCAGCTGGGTGTCTTGCAGGCCGTC | CTCAACTGGTGTCGTGGAGTCGGCAATTCAGTTGAGTGCATG |
| hsa_miR_ 545 | ACACTCCAGCTGGGTCAGTAAATGTTTATTAG | CTCAACTGGTGTCGTGGAGTCGGCAATTCAGTTGAGTCATCT |
| hsa_miR_548p | ACACTCCAGCTGGGTAGCAAAAACTGCAGTT | CTCAACTGGTGTCGTGGAGTCGGCAATTCAGTTGAGAAAGTA |
| hsa_miR_564 | ACACTCCAGCTGGGAGGCACGGTGTCAGCAG | CTCAACTGGTGTCGTGGAGTCGGCAATTCAGTTGAGGCCTGC |
| hsa_miR_567 | ACACTCCAGCTGGGAGTATGTTCTTCCAGGAC | CTCAACTGGTGTCGTGGAGTCGGCAATTCAGTTGAGGTTCTG |
| hsa_miR_568 | ACACTCCAGCTGGGATGTATAAATGTATAC | CTCAACTGGTGTCGTGGAGTCGGCAATTCAGTTGAGGTGTGT |
| hsa_miR_ 578 | ACACTCCAGCTGGGCTTCTTGTGCTCTAGG | CTCAACTGGTGTCGTGGAGTCGGCAATTCAGTTGAGACAATC |
| hsa_miR_ 605 | ACACTCCAGCTGGGTAAATCCCATGGTGCC | CTCAACTGGTGTCGTGGAGTCGGCAATTCAGTTGAGAGGAGA |
| hsa_miR_607 | ACACTCCAGCTGGGGTTCAAATCCAGATCT | CTCAACTGGTGTCGTGGAGTCGGCAATTCAGTTGAGGTTATA |
| hsa_miR_ 643 | ACACTCCAGCTGGGACTTGTATGCTAGCTC | CTCAACTGGTGTCGTGGAGTCGGCAATTCAGTTGAGCTACCT |
| hsa_miR_665 | ACACTCCAGCTGGGACCAGGAGGCTGAGGC | CTCAACTGGTGTCGTGGAGTCGGCAATTCAGTTGAGAGGGGC |
| hsa_miR_668 | ACACTCCAGCTGGGTGCGCCTCGGGTGAGC | CTCAACTGGTGTCGTGGAGTCGGCAATTCAGTTGAGCATGCT |
| hsa_miR_578 | ACACTCCAGCTGGGCTTCTTGTGCTCTAGG | CTCAACTGGTGTCGTGGAGTCGGCAATTCAGTTGAGACAATC |

R: TGGTGTCGTGGAGTCG
